# Idiopathic Intracranial Hypertension: A Case Report

**DOI:** 10.31729/jnma.5176

**Published:** 2021-02-28

**Authors:** Anupam Ghimire, Achal Raj Acharya, Anish Karn, Mukesh Kumar Jha

**Affiliations:** 1 Department of General Practice and Emergency Medicine, Patan Academy of Health Sciences, Lagankhel, Lalitpur, Nepal; 2 Department of General Medicine, Nobel Hospital, Sinamangal, Kathmandu, Nepal; 3 Department of Ophthalmology, Patan Academy of Health Sciences, Lagankhel, Lalitpur, Nepal

**Keywords:** *acetazolamide*, *idiopathic intracranial hypertension*, *pseudotumor cerebri*

## Abstract

Idiopathic Intracranial Hypertension (IIH) is a rare occurrence in young, physically fit male and a diagnosis of exclusion among most patients presenting with signs and symptoms of raised intracranial pressure. Here we describe a case of a young male in the ideal weight range with no previous exposure to offending chemicals presented with a history of headache, obscuration of vision, and photopsia. On examination, there were no positive neurological findings. Increased opening pressure was found on the lumbar puncture. Ophthalmological examination revealed bilateral papilledema. Humphrey's Visual field test showed peripheral field loss. MRI scan of the brain and orbits were normal. The patient was diagnosed and managed in primary care setting after neurosurgical consultation. Though rare, we should suspect IIH in ideal body weighted male if the headache is persistent after other causes of headache have been ruled out.

## INTRODUCTION

Idiopathic intracranial hypertension (IIH), also known as pseudotumor cerebri, is a disorder characterized by increased intracranial pressure (ICP) of unclear pathogenesis, with an absence of intracranial mass lesions or cerebrospinal fluid (CSF) outflow obstruction.^1,2^ It occurs in about one to two per 100,000 people, with severe visual loss in 10-30% of patients.^3-6^ It occurs most commonly in females of childbearing age women and increased risk among obese.^6-8^ We report a case of a young male with ideal weight range without comorbidity presented with intractable headache and blurred vision later diagnosed as IIH.

## CASE REPORT

A previously healthy thirty-one-year-old male employed in the recycling industry presented with a headache history for two weeks. It was throbbing and pulsatile nature that was most prominent in the bilateral temporal regions. The severity was enough to hamper his sleep and daily activities. The headache was associated with the obscuration of both eyes' vision for around a minute several times per day. He also perceived brief flashes and floaters in both eyes without the presence of light. He was a non-hypertensive, euthyroid and did not have tuberculosis and Diabetes Mellitus. There was no history of trauma, loss of consciousness, fever, vomiting, shortness of breath, tinnitus, hemoptysis, and weight loss. He denied any changes in his bowel and bladder habits. There is no significant family history.

**Figure 1.**
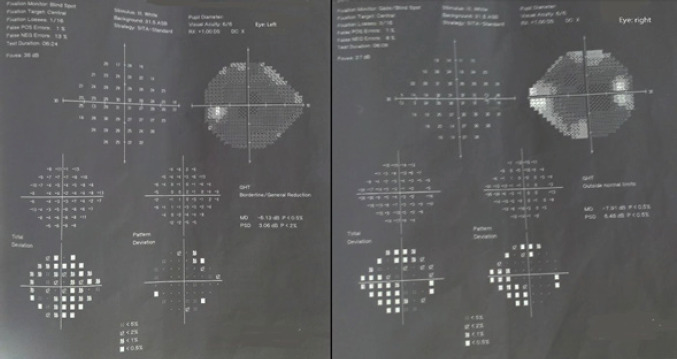
Humphrey's visual field tests revealed the pattern of vision loss in the left eye (left) and the right eye (right).

Vitals and physical examinations were normal. Bulk, tone, and power of all limbs were normal, and all reflexes including Babinski's reflexes that were bilaterally down going. Ophthalmologic examination revealed normal visual acuity. The anterior segment examination showed normal findings with both direct and consensual light reflex present with the absence of a relative afferent pupillary defect. The color vision is normal in both eyes. The retinoscopy findings showed +0.25 dioptre power in both eyes. The fundoscopy examination revealed bilateral optic disc edema with hyperemia. Humphrey's Visual field test (Figure 1) was performed that showed a peripheral visual field loss in both eyes.

Lumbar puncture was performed, and increased opening pressure (>60 mmHg) was noted. After the procedure, he reported a reduction in the severity of symptoms. The CSF report was normal. The hematological test and thyroid function test were normal. Magnetic resonance imaging of the brain and orbits were normal. Later, an electroencephalogram was done, which was unremarkable except that waves were slower in speed than expected in his age group. The patient was managed with volume reducing agents (acetazolamide), antiepileptics, analgesics, and muscle relaxants. He is on regular follow-up visits and has been responding well to the treatment.

## DISCUSSION

Our report describes a young male patient diagnosed with IIH, although exact mechanisms are unclear. Few theories debate on its pathogenesis: (1) an increase in production of CSF; (2) a higher levels of cerebral blood flow leading to an increased fluid content and (3) a restriction in venous drainage.^10^

The most common clinical features include headache, usually of a throbbing nature associated with nausea and vomiting triggered by coughing, sneezing, or straining. Other symptoms include pulsatile tinnitus and transient visual obscuration.^6,8^ The IIHT trial reported headaches among 84% of participants at presentation.^4,8^ Mostly, abducens nerves are affected, resulting in horizontal nystagmus. Occasionally the facial nerve is affected, producing palsies on the bilateral face. Another highly debilitating effect of IIH is papilledema, swelling of the optic disc. Not all patients experience papilledema symptoms, which, if present, is reported as obscuration of vision. Long-term papilledema leads to peripheral visual loss initially and progressively. It encompasses a central vision which Humphrey's visual field testing can map.^9^ The modified Dandy criteria remains the most appropriate till date for diagnosis of the disorder. It applies as:^7-9^

1. Symptoms and signs of increased intracranial pressure (headache, transient visual obscuration, pulse synchronous tinnitus, papilledema, visual loss)
2. No other neurologic abnormalities or impaired level of consciousness
3. Elevated intracranial pressure with normal CSF composition
4. A neuroimaging study that shows no etiology for intracranial hypertension
5. No other cause of intracranial hypertension apparent

Neuroimaging (Magnetic Resonance Imaging and Non-Contrast Computed Tomography of the brain and orbits) and CSF analysis are required to exclude other causes of raised intracranial pressure, whereas for assessing the severity of vision and papilledema, ophthalmological evaluation is needed.^8,9^

Management is mainly targeted to protect vision and next aimed to alleviate symptoms.^7,8^ Lumbar puncture is the first line of treatment in impending vision loss due to raised CSF pressure. Carbonic anhydrase inhibitors such as acetazolamide remain mainstay medical treatment and have been widely and successfully used.^9^ In IIHTT, the use of acetazolamide with low sodium and weight reduction diet brought significant improvement in visual function. Most of the participants responded well to doses of 500 mg to 1gm twice a day while it was increased to a maximum dose of 2gm twice daily.^6,9,10^ Caution should be taken in patients with low potassium levels.^9^ Alternately, are prescribed furosemide, although its effectiveness is significantly lower. Low dose antidepressants and antiepileptics such as amitriptyline and topiramate are used respectively for pain management as well as prevention of seizures.^5,6^

Patients not responding to medical therapy are usually recommended to transverse sinus venous stenting which led to a high correction rate of 87%.^6,10^ Further improvements in approach and a better understanding of the pathogenesis is required to improve outcomes in those with post-procedure complications in which 1-2% of people suffer blindness.^9-10^

IIH should be strongly suspected in women of childbearing age with higher body mass index (BMI). However, if a man presents with typical features of IIH, despite the low incidence among males and with low BMI, IIH should be suspected clinically. Though rare, we should suspect it if headache presents with obscuration of vision. Radiological investigation and ophthalmological evaluation should always be performed to rule out other anatomical and obstructive causes of raised ICP. This patient was diagnosed and managed in a primary care facility. An earlier diagnosis may make the patient pain free and encourage good quality of life.
